# Stakeholder perspectives on adaptive clinical trials: a scoping review

**DOI:** 10.1186/s13063-020-04466-0

**Published:** 2020-06-17

**Authors:** Tina Madani Kia, John C. Marshall, Srinivas Murthy

**Affiliations:** 1 BC Children’s Hospital Research Institute, 4500 Oak Street, Vancouver, BC Canada; 2 Li Ka Shing Knowledge Institute, Unity Health Toronto, University of Toronto, Toronto, ON Canada

**Keywords:** Adaptive clinical trials, Adaptive designs, Bayesian statistics

## Abstract

**Background:**

Adaptive clinical trials (ACTs) represent an emerging approach to trial design where accumulating data are used to make decisions about future conduct. Adaptations can include comparisons of multiple dose tiers, response-adaptive randomization, sample size re-estimation, and efficacy/futility stopping rules. The objective of this scoping review is to assess stakeholder attitudes, perspectives, and understanding of adaptive trials.

**Methods:**

We conducted a review of articles examining stakeholders encompassing the broad medical trial community’s perspectives of adaptive designs (ADs). A computerized search was conducted of four electronic databases with relevant search terms. Following screening of articles, the primary findings of each included article were coded for study design, population studied, purpose, and primary implications.

**Results:**

Our team retrieved 167 peer-reviewed titles in total from the database search and 5 additional titles through searching web-based search engines for gray literature. Of those 172 titles, 152 were non-duplicate citations. Of these, 119 were not given full-text reviews, as their titles and abstracts indicated that they did not meet the inclusion criteria. Thirty-three articles were carefully examined for relevance, and of those, 18 were chosen to be part of the analysis; the other 15 were excluded, as they were not relevant upon closer inspection.

Perceived advantages to ADs included limiting ineffective treatments and efficiency in answering the research question; −perceived barriers included insufficient sample size for secondary outcomes, challenges of consent, potential for bias, risk of type 1 error, cost and time to adaptively design trials, unclear rationales for using Ads, and, most importantly, a lack of education regarding ADs among stakeholders within the clinical trial community. Perceptions among different types of stakeholders varied from sector to sector, with patient perspectives being noticeably absent from the literature.

**Conclusion:**

There are diverse perceptions regarding ADs among stakeholders. Further training, guidelines, and toolkits on the proper use of ADs are needed at all levels to overcome many of these perceived barriers. While education for principal investigators is important, it is also crucial to educate other groups in the community, such as patients, as well as clinicians and staff involved in their daily implementation.

## Background

Fixed randomized clinical trials are considered the current gold standard for evaluating the efficacy of novel treatments, where the anticipated effect size and the estimated event rate in the control group are used to determine a fixed sample size. Adaptive clinical trials (ACTs), on the other hand, represent an emerging approach to trial design and conduct where accumulating data within a trial are used to make decisions about the trial’s future conduct [1]. Adaptations can include changing multiple dose tiers, response-adaptive randomization, sample size re-estimation, and altering efficacy/futility stopping thresholds [2]. Adaptive designs (ADs) lend a certain level of flexibility that can allow researchers to answer research questions more efficiently while reducing trial size and duration. ACTs with response-adaptive randomization can also have potential ethical benefits, with more patients potentially being assigned to more successful treatments.

ADs have become increasingly discussed in recent years. The Pharmaceutical Research and Manufacturers of America (PhRMA) and the Biotechnology Industry Organization (BIO) have established AD working groups, methodologies, and implementations for regulatory considerations [3]. However, there are concerns regarding a potential for increased risk of type I error and bias, and the complexity of such studies [4].

There are highly diverse opinions about the utility, efficiency, understanding, and acceptance of ADs among members of the clinical trial community [5]. We conducted a scoping review of available evidence in order to examine stakeholder perspectives of ADs. By gaining a better understanding of current opinions regarding ADs, we seek to make recommendations to researchers hoping to conduct such trials and to the broader research community.

## Methods

The term “stakeholders” encompasses the broad clinical trial community and includes, but is not limited to, physicians, researchers, statisticians, review board members, patients, and their families/advocates. In order to fulfill our objectives, we conducted a scoping review of literature published between 1980 and 2019 regarding stakeholder perspectives on ADs. We picked 1980 as the start date, as this is when the concept of adaptive randomization began to be prominently discussed and explored.

### Protocol

The protocol for this scoping review was based on established frameworks [6].

### Eligibility criteria

For the purposes of this scoping review, full-length empirical research articles and gray literature regarding ADs or ACTs were chosen. The inclusion criteria are:

1. Articles published between 1980 and 2019
2. English language articles or experiences disseminated by peer-reviewed journals, regulatory agencies, meeting reports, or companies involved in trials
3. Articles or reports that have surveyed individuals or retrospectively collected data regarding perspectives on ADs or ACTs.

### Search strategy and information sources

To identify relevant studies and the gray literature, a computerized search was conducted by the lead author (TM) of MEDLINE, Embase, and the University of British Columbia library databases. We selected search terms that would allow us to find articles that look at perspectives or attitudes of various stakeholders (Supplementary Table 1). The gray literature was surveyed through the web-based search engine Google Scholar using the same search terms.

### Study selection

Titles and abstracts of all publications were imported into Mendeley software by TM. The articles were screened for relevance, and full articles and reports were closely examined to determine whether they met all predetermined inclusion and exclusion criteria, with disagreements resolved by consensus.

### Charting and study synthesis

Following review of articles, the primary findings of each article were coded for study design, population studied, purpose, and primary implications (Table 1), which were entered into a data charting form by TM. Primary implications were later categorized by hand as either perceived facilitators or barriers to AD use through a narrative thematic analysis.

**Table 1:** Characteristics of included studies

Lead author and date	Population	Study design	Main findings
Benda et al. 2010 [7]	International representatives from industry, academia, and regulatory agencies	Statistical considerations and issues session conference proceedings	-Adaptive designs (ADs) should ensure the integrity of a trial and the validity of its conclusions -Adaptivity is a fundamentally important concept that can be applied to many different stages of drug discovery and development
Chappell et al. 2017 [8]	Researchers and representatives from industry	Conference proceedings on statistical issues in clinical trials	-Grants for developing a response-adaptive randomization design are crucial -There are ethical considerations that arise with respect to response-adaptive randomization
Coffey et al. 2012 [9]	Representatives from the National Institutes of Health (NIH), the Food and Drug Administration (FDA), the European Medicines Agency (EMA), the pharmaceutical industry, non-profit foundations, the patient advocacy community, and academia	Recommendations from a workshop	**6 recommendations:** -Need for a better-defined taxonomy of the framework for ADs -Need to better quantify the statistical risks (e.g., statistical bias, potential increase in type I error rates, and risk for covariate imbalance) -Better understanding of the concept of “adaptive by design” -NIH should offer more recognition and funding for planning clinical trials that might benefit from ADs -Use of ADs may require a different way of thinking about the structure and conduct of data safety monitoring branches -Need for education of the clinical trials community regarding the use of ADs
DellaCioppa 2013 [10]	Senior executives from more than 20 companies, including 11 of the top 15 pharmaceutical companies	Executive roundtable on AD trials	-AD needs to be even more widely adopted across industry -Simple ADs should be considered part of Good Clinical Practice
Dibao-Dina et al. 2018 [11]	International Review Boards members	Web-based survey via Sphinx Survey, clinical vignettes	-The most important ethical justifications for unequal randomization are gaining experience in treatment and reducing drop-outs -Different definitions of equipoise exist which may have caused the discrepancy when determining ethics of adaptive clinical trials (ACTs)
Dimairo et al. 2015a [12]	Key stakeholders in clinical trials research (Clinical Trials Unit [CTU] directors, funding board and panel members, statisticians, regulators, chief investigators, data monitoring committee members, and health economists)	Semi-structured, in-depth interviews of key stakeholders	**5 major barriers identified:** -Lack of practical knowledge and applied training with insufficient case studies -Time constraints for planning -Lack of awareness of AD opportunities or acceptable scope -Statistical and operational complexities -Conservatism regarding use of ADs **Recommendations:** -Accessible publications of successful and unsuccessful trials -Toolkit for ACT design
Dimairo et al. 2015b [13]	Key research stakeholders—predominantly in the UK, CTUs, public funders, and private sector organizations	Cross-sectional, online parallel surveys	-Top-ranked barriers toward using ACTs: lack of bridge funding to support design work of complex AD, lack of practical implementation knowledge and hands-on experience, researcher providing inadequate rationale for use of ACTs -Need exists for a guidance document or troubleshooting kit regarding ACT design and implementation
Elsäßer et al. 2014 [14]	EMA	Text search of scientific advice letters regarding ADs	**Questions that are generally addressed by assessors when evaluating adaptive clinical trial proposals:** -Is there a good rationale? Have alternative, more standard trial designs been considered? -Does the proposal fit well in the context of the development program? -Can the proposal be implemented without important damage to trial integrity? -Is the type I error rate controlled? -Has the potential bias of treatment effect estimates been evaluated? -Is the proposal practical and feasible?
Food and Drug Administration 2018 [15]	Food and Drug Administration (FDA or Agency) on ADs	The draft guidance to represent the current thinking of the FDA	**Advantages of ADs include:** -Statistical efficiency -Ethical considerations -Advantages in generalizability and improved understanding of drug effect -Acceptability to stakeholders **Barriers include:** -Complex analytical methods required -Gains in efficiency in some respects may be offset by losses in other respects -Efficiency gains may be limited in certain clinical settings -An adaptive change to a trial design may lead to results after the adaptation that are not similar to those before the adaptation
Guetterman et al. 2015 [16]	Statisticians, clinician researchers, and representatives from the FDA and NIH who took part in the ADAPT-IT program	Qualitative study via telephone using semi-structured interview protocol	-Participants thought ADs could increase efficiency of trials and decrease costs -Indicates need to educate the broader research community and more training resources to continue learning
Hartford et al. 2018 [17]	Pharmaceutical companies, academic institutions, and contract research organizations	Survey	-Stopping early for safety and changing the endpoint of the analyses were rarely mentioned in literature prior to 2012 but are now appearing more frequently -The barriers of change management and negative experiences by some institutions with ADs remain a source of concern -Consistent training would be helpful to choose the right adaptation(s) -Perceived barrier of regulatory acceptance also remains a concern, which could be alleviated by an update of the FDA draft guidance to industry on ADs
Jaki 2013 [18]	UK CTUs	Survey	**Barriers to using ADs:** -Lack of expertise -Lack of time -Lack of software -Lack of funding structure -Investigator insistence on traditional designs
Legocki et al. 2015 [19]	Clinical trial experts: academic biostatisticians, consultant biostatisticians, academic clinicians, and other stakeholders including patient advocacy, NIH, and FDA representatives	Self-administered ACT beliefs survey, open-ended questions, mini-focus groups	-Benefits of ACTs include higher probability of receiving an effective intervention, optimizing resource utilization, and accelerating treatment discovery -Disadvantages of ACTs include possible bias and lack of informed consent
Lin et al. 2016 [20]	Center for Biologics Evaluation and Research	Retrospective survey within Center for Biologics Evaluation and Research	**The following questions should be asked before utilizing ADs:** -Why use ADs? -What features does proposed design have and are they clear in protocol? -Is type I error controlled? -Are the simulation studies adequate? -Is operational bias minimized? -Are success criteria and stopping rules specified?
Meurer et al. 2016 [21]	Clinicians, preclinical scientists, and biostatisticians who were planning clinical trials and part of the ADAPT-IT program	Visual analog scale (VAS) items through paper survey/web-based survey, mini-focus groups	-There is a need for greater community education regarding ACTs as many think clinicians don’t understand it -ACTs can help reduce sample size needed for studies but may limit assessment of secondary outcomes
Morgan et al. 2014 [22]	92 organizations worldwide inclusive of industry (pharma, biotech, contract research organizations [CROs]) and academia	Web-based survey	**Barriers to ADs include:** -Lack of education about design leads to uncertainties about methodology -Time management and planning for simulations -Risk of regulatory acceptance -Academia has a lack of resources for modeling
Quinlan et al. 2010 [23]	13 large- and medium-sized pharmaceutical companies and 3 statistical consultancies	Survey	**5 main barriers to ADs:** -Requirement of additional planning time -Willingness of the project team to engage in the additional activities and simulations -Availability of statistical and clinical expertise and software tools -Impact of adaptive approaches to functional lines supporting clinical development -Insufficient top-down financial and motivational support from the R&D organizations
Wang 2010 [24]	A panel with international representatives from industry, academia, and regulatory agencies	Conference proceedings titled “On “Perspectives on the Use of Adaptive Designs in Clinical Trials”	**Principal statistical issues of an AD include:** -The probability of correct selection in lieu of type I error -Type II error in exploratory adaptive trials -Committing a type I error in confirmatory adaptive trials -Bias in treatment effect estimates and its related issues with the interpretability of a positive study result due to adaptation

## Results

**Fig. 1:**
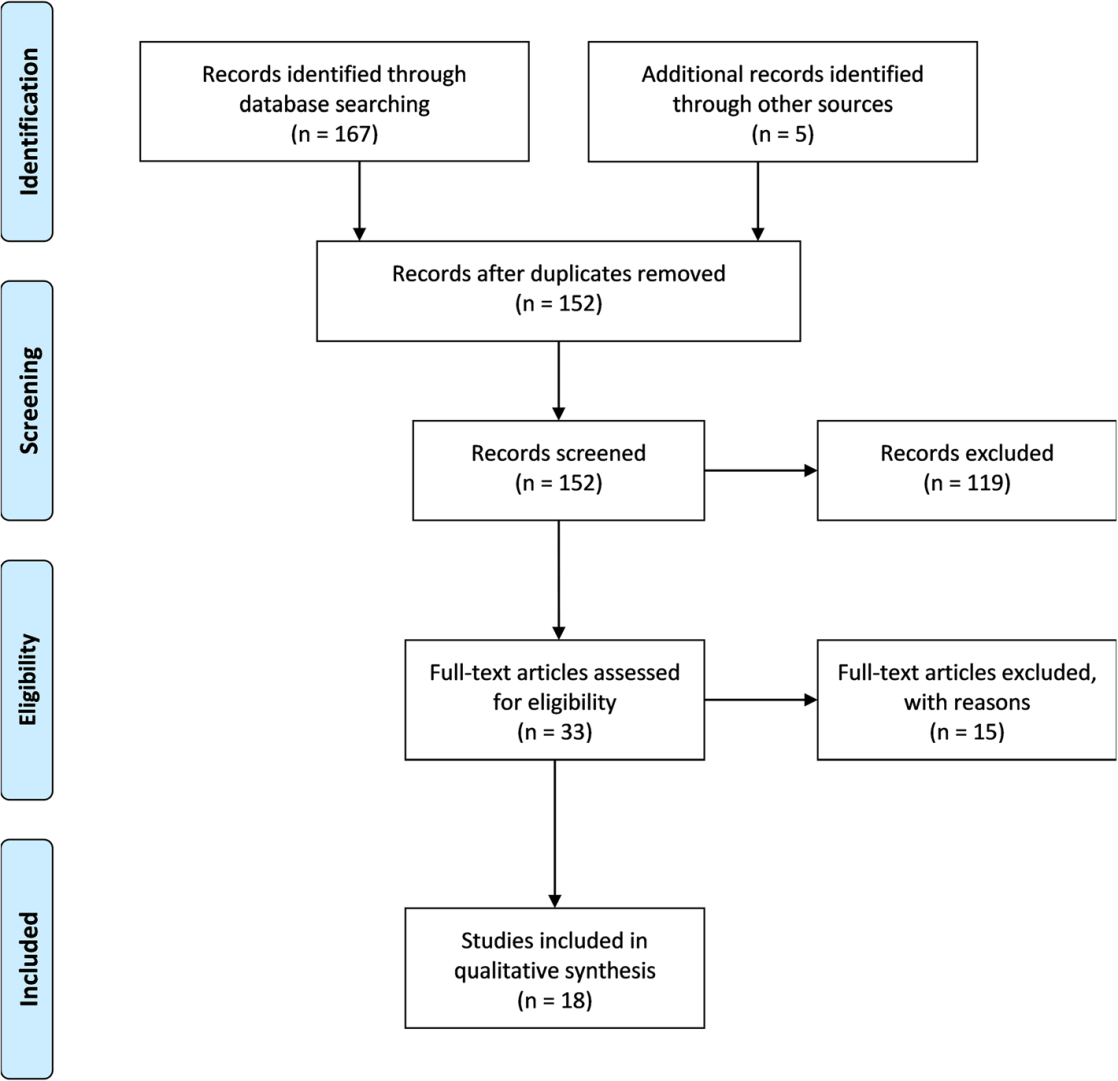
Flowchart of screening process

We retrieved 167 titles from the database search. Of these, 152 were non-duplicate citations (Fig. 1). Of these, 119 were not given full-text reviews, as their titles and abstracts indicated that they did not meet the inclusion criteria. Thirty-three articles were reviewed in full, and of those, 18 met inclusion criteria and were chosen by TM to be part of the analysis (Table 1), with the remaining 15 excluded; they were not relevant upon closer inspection, as they were theoretical or statistical discussions rather than surveys of stakeholders. Supplementary Tables 2 and 3 list the titles and the full-text articles excluded for not meeting inclusion criteria.

## Thematic analysis

Participants’ views can be categorized into lists of perceived facilitators and barriers to ADs when compared to traditional trials:

1. Summary of facilitators to ADs

- Efficiency in design
- Limiting ineffective treatments

2. Summary of barriers to ADs

- Insufficient sample size for secondary outcomes
- Bias due to unblinding
- Informed consent
- Type I error inflation
- Confusion over terms
- Clear rationale for using ADs
- Time taken to design ADs
- Cost: need for further grants and funding structures
- Lack of education

### Facilitators

#### Limiting patient exposure to ineffective treatments

The FDA lists ethical considerations in early stopping for studies with ineffective treatments as a primary advantage of ADs [15]. Furthermore, three studies stated that a facilitator of ADs was limiting the number of patients who were exposed to ineffective treatments [12, 16, 19]. Two studies found that clinical trialists believe response-adaptive randomization gives patients a better chance at being randomized to the better arm in a clinical trial [12, 19]. A participant in one of the studies [12] stated, “the biggest opportunity is stopping poor drugs early, most of our drugs fail…we should be killing those drugs.” Participants in the ADAPT-IT education program, which aimed to educate researchers about adaptive trials, stated that ADs provide a tool for researchers to identify successful treatments and terminate futile treatments [16]. An executive roundtable on ADs also shared this view, stating that evidence from leading companies show that stopping for futility saves upwards of $100 million per annum when applied at the portfolio level [10].

#### Efficiency in answering research questions

Focus groups from one publication found that most clinicians and non-statistician researchers believe that ADs can reduce the overall sample size of a trial [21]. While the biostatisticians believed this efficiency could cut the costs associated with running clinical trials, they were unsure if ADs can successfully reduce study sample size, as it is dependent on the phase of the trial, what is being adapted, and how the researchers define efficiency. In the opinion of the academic biostatisticians, a comparison of a trial completed under different designs was needed to answer the question of what advantages AD trials conferred. They did think that ADs can improve efficiency by increasing the number of scientific questions that can be answered in certain kinds of adaptive trials. Most participants in another study shared this view, as they stated that ADs could increase efficiency by allowing researchers to answer a number of questions in a single study, therefore reducing costs and resources used [16]. Stakeholders interviewed in a third study listed efficiency in trial design and increased value for money as two of the three major benefits to ADs, as they potentially allow quicker answering of research questions [13]. They also believed ADs make efficient use of available resources, as stopping trials early means resources are reallocated to other important areas [13]. Statistical efficiency in answering research questions was also listed as an advantage to ADs in the FDA’s most recent regulatory guidance document [15].

### Barriers

#### Sufficient sample size for secondary outcomes

Results of one survey recommended researchers to be wary of stopping studies too early, as the sample size may not be large enough to assess secondary endpoints, in particular if the secondary endpoint has less frequency, such as mortality [20]. The same opinion was shared by the participants in another study, stating that researchers may lose the power with which to study secondary outcomes if, for example, too few patients are assigned to the “worse” arm, reinforcing the need for very careful outcome selection and trial planning [21].

#### Time taken for design

While some previously mentioned studies concluded that ADs can be advantageous in terms of answering the research question more quickly and reducing time spent on the study in total, another study revealed that time constraints to designing ADs relative to traditional designs were reported as a barrier by 48% of respondents [13]. Another study surveying Clinical Trials Units (CTUs) in the UK found that CTU members felt that the current funding structure often leaves insufficient time to explore the innovative range of options and plan adaptive studies carefully [18]. A summary of findings from a survey of pharmaceutical companies conducted through PhRMA’s working group on ADs further listed additional planning time as one of the five main barriers to conducting adaptive studies [23]. The FDA’s 2018 draft guidance on ADs also states that preplanning AD modifications can require more effort at the design stage, leading to longer lead times between planning and starting the trial [15].

#### Informed consent

Consent was mentioned in two studies, with academic biostatisticians in one study suggesting that achieving informed consent in ADs can become complicated [19]. Another study mentioned that board reviewers have different working definitions of the term “equipoise” and therefore have varying opinions regarding the ethics of ADs [11].

#### Bias due to unblinding

In mini-focus group discussions held among biostatisticians in one study, participants were concerned about the risk of unintentional unblinding leading to selection bias during the enrollment period in a trial [19]. Consultant biostatisticians also believed operational bias to be an issue, especially if “appropriate firewalls” are not in place. A summary of panel insights on ADs states that operational bias can be a result of changes in trial conduct due to preplanned adaptations, or in trial implementation due to monitoring by the unblinded parties who either have scientific interests or financial interests, among other factors impacted by unblinding [24]. While this bias can be minimized by separating the professionals who have access to unblinded interim data from those who implement the adaptation decisions, such separation must be carefully balanced against the risk of decreasing the quality of data management, analysis, and decision-making [7].

#### Type I error inflation

Three other studies and one panel discussion revealed risk of type I error inflation to also be a barrier [7, 9, 14, 20]. The justification for a type I error concern as discussed in one panel was that “simulations can only provide type I error rates for a limited number of parameter configurations…with complex designs or complex models it is often unclear under which parameter configurations the type I error rate is maximal.” [6]. Furthermore, with complex ADs, type I error rate control is usually unable to be predicted mathematically. In these cases, researchers are tasked with considering alternatives to a purely simulation-based approach [7].

#### Clear rationale for using adaptive designs

Another very common theme was the need for adaptive research proposals to clearly outline their rationale for choosing ADs and how this will help answer the research question. One publication’s interviews with clinical trialists found that regulatory disapproval is mostly due to inadequate description of the proposed AD and its suitability to address the research question [11]. In their subsequent cross-sector surveys of CTUs, public funders, and private organizations, researchers’ inadequate description of the rationale for using an AD was reported as an ”at least moderately” important barrier to approving adaptive studies by 60% of UK public funders [13]. A meeting regarding the statistical considerations of ADs with international representatives from industry, academia, and regulatory agencies also stated that the written charter should be well documented to mitigate bias issues and to confirm trial integrity [24].

Two other studies also concluded that carefully assessing the potential advantages and disadvantages of an AD over a traditional trial design at the study planning stage is important, especially for phase III confirmatory trials [14, 20]. One study stated that trialists should consider if simple, conventional ADs, such as group sequential methods, will meet their needs over complex designs [20], while the other recommended considering alternate standard designs and evaluating one’s rationale for using ADs [14].

#### Confusion over terms

One study found that stakeholders remain confused about the definition of ADs. They acknowledged a broadening of the definition over the past few years, with the term having been loosely defined in the first place. Thus, a great deal of confusion still exists in the general scientific community, and future discussion is needed to better clarify what distinguishes one type of AD from another [12].

#### Cost

Multiple studies outlined the need for further funding, with one recommending further National Institutes of Health (NIH)-funded grants for ADs [9] and another identifying the need for better funding structures in CTUs for trial planning [18]. In a panel discussion regarding statistical issues in clinical trials, one researcher spoke of the benefits of grants for developing a response-adaptive randomization design, stating, “These grants could provide a year of funding to conduct the simulation studies and work through the issues prior to the start of the trial. Continued funding of methodology grants is also necessary in order to further the science because there are still a lot of questions to be answered in this area.” [8]. Another survey of pharmaceutical companies listed a major barrier to ADs being insufficient top-down financial and motivational support from Research & Development organizations to build a scalable infrastructure [23].

#### Lack of education and resources

A topic that was mentioned by eight studies was the need for further education and understanding of ACTs among physicians, researchers, and reviewers. One study found the lack of applied training and insufficient access to case studies on ADs to be top-ranked barriers [13]. The study indicated the need for accessible publications of successful (as well as unsuccessful) AD case studies and regional focal groups of experts to support those CTUs wishing to implement ADs. Furthermore, they encourage researchers who receive public funding for AD-related methodological research to produce open access resources to implement the methods developed. Other studies recommended practical education tailored to clinical trialists such as educational seminars and practice-oriented workshops, which can facilitate translational knowledge sharing [12] and further resources for modeling adaptive trials [22]. A survey of pharmaceutical companies and statistical consultancies as well as the FDA’s latest draft guidance on ADs also state lack of software tools and statistical expertise as a major barrier to AD implementation [15, 23].

Likewise, four other studies [9, 16, 17, 22] revealed the lack of education regarding ADs as a main barrier to their usage. Participants in another study expressed similar concerns that clinicians and clinician-researchers have limited understanding of ADs [21]. They noted that although AD trials are becoming more common, those in academic settings have relatively little experience designing and conducting ADs. Thus, there was also the concern that, when research results are published, the broader medical community would have very little understanding of the actual design.

Finally, one study showed that uncertainty also exists regarding the ability of review boards and organizations such as the NIH and FDA to understand and accept ADs [21]. They cited the NIH’s short page limits and the challenge of understanding the complex designs, particularly with a reduced ability to reproduce the sample size estimations compared to fixed designs. In general, the participants perceived that the FDA would have the greatest understanding of ADs; however, they also felt that the FDA desires simple designs so that they can unequivocally support the primary outcome measure and so practitioners can easily understand the results. Another study suggested a periodic “refresher training” of public funding boards and panel members prior to their commissioning meeting which may help alleviate a lack of awareness of the acceptable scope of ADs [12]. A third study recommended that the NIH develop programs for educating and training researchers, reviewers, and data safety and monitoring board members about potential areas that might benefit from the use of ADs [9].

### Inter-sector perceptions

As a variety of different types of stakeholders were surveyed, opinions did vary between groups of individuals. One study found that complexities during practical implementation, inadequate data management infrastructure, and fear of risking regulatory approval appear to be very prominent concerns in the private sector. In contrast, the lack of bridge funding to support developmental design work and worry about research staff employment contracts when trials are stopped early were highly and middle rated in the public sector, respectively [13].

A study surveying institutional review boards found physicians surveyed to be skeptical about the legitimacy of unbalanced randomization, especially for cost and ethical issues. Philosophers and ethicists were also opposed to reasons of cost and ethical issues but were believers of unbalanced randomization for safety and methodological reasons only if it was scientifically required [11].

In a study surveying the perspectives of clinicians, biostatisticians, and other stakeholders, the academic clinicians and other stakeholders had roughly similar patterns of rankings of the ethical advantages and disadvantages of ACTs [19]. The consultant biostatisticians took positions similar to those of the academic clinicians and other stakeholders, although their ratings more strongly emphasized the ethical advantages. On the other hand, the academic biostatisticians had some overlap with the academic clinicians and other stakeholders, but their positions de-emphasized ethical advantages. Over all, inter-sector differences in this study were greatest between the academic biostatisticians and the consulting biostatisticians, as they rated oppositely on five of the six ratings.

## Discussion

In this scoping review, we summarized the available literature on perspectives of the clinical trial community regarding ADs and ACTs. We found that stakeholders perceive more barriers than facilitators to ADs. We do not attempt to determine whether these barriers or facilitators are “right,” given that there is no way of definitively answering that question; what we do examine is the perceptions of these designs among the larger community. Many of these perspectives reflect a stated need for further education of the research community regarding ADs. As they become more common designs, addressing these perceptions among the stakeholder community is crucial for their successful implementation.

The 18 articles included in this scoping review addressed issues relevant to perspectives of physicians, statisticians, researchers, ethicists, review board members, regulatory organizations, and industry such as pharmaceutical companies. Notably, a gap in the literature exists in terms of patient perspectives on ADs, as no studies found had interviewed patients. Furthermore, ethics review board members were only surveyed about unequal randomization in the available literature, not ADs or ACTs more broadly. Therefore, further studies are needed to fill gaps regarding patient and review board member perspectives on clinical trials, given their integral role. Future work in this area should explore what kinds of guiding frameworks stakeholders would find most useful. While education for principal investigators is important, it is crucial to educate other groups in the community as well, e.g., clinicians and staff involved in their daily implementation. Further studies looking into specific gaps in knowledge for different types of roles in the clinical trial community are also needed.

Furthermore, there may be a lack of clarity on definitions of an AD. The FDA’s 2018 draft guidance on ADs classifies an AD as a “clinical trial design that allows for prospectively planned modifications to one or more aspects of the design based on accumulating data from subjects in the trial” [15]. However, frequentist analyses with sample size re-estimation fit these criteria, as well as trials with a full Bayesian analysis. At the same time, many believe Bayesian studies to be the only type of AD. Therefore, to limit confusion, studies involving perspectives on ADs should clarify their actual working definition of ADs. Given the diversity in definition, many of the barriers and facilitators may apply in different ways across the AD spectrum.

This study is limited by our search terms which, though selected by authors after careful consideration to include a broad scope of terms used in the literature, could have failed to capture relevant peer-reviewed articles or gray literature. Thematic analyses were performed post hoc and do not account for limitations of the specific studies regarding representativeness of sampled populations or flaws in the evaluation tools used in the included studies.

## Conclusion

The implementation of innovative clinical trials in practice is always fraught with challenges. This review indicated that there are major perceived facilitators to ADs such as limiting ineffective treatments and efficiency in answering the research question, as well as many perceived barriers, including insufficient sample size for secondary outcomes, issues of consent, potential for bias, cost and time to adaptively design trials, unclear rationales, and most importantly, a lack of education among stakeholders within the clinical trial community. Given their increasing frequency, further training, guidelines, and toolkits about the proper use of ADs are needed at all levels to overcome many of these perceived barriers and to better inform implementation among all relevant stakeholders.

## Supplementary information

**Additional file 1:Supplementary Table 1.** Search terms. **Supplementary Table 2.** List of records screened and excluded due to not meeting inclusion criteria. **Supplementary Table 3.** List of full-text articles assessed but excluded due to not meeting inclusion criteria.

## Data Availability

All data generated or analyzed during this study are referenced in this published article.

## Abbreviations

ACT: Adaptive clinical trial
AD: Adaptive design
BIO: Biotechnology Industry Organization
CIHR: Canadian Institutes of Health Research
FDA: Food and Drug Administration
NIH: National Institutes of Health
PhRMA: Pharmaceutical Research and Manufacturers of America

## References

[1] Sibbald B, Roland M (1998). Understanding controlled trials: why are randomised controlled trials important?. BMJ..

[2] Dragalin V (2006). Adaptive designs: terminology and classification. Drug Inf J.

[3] Gallo P, Chuang-Stein C, Dragalin V, Gaydos B, Krams M, Pinheiro J (2006). Adaptive designs in clinical drug development—an Executive Summary of the PhRMA Working Group. J Biopharm Stat..

[4] Bothwell L, Kesselheim A (2017). The real-world ethics of adaptive-design clinical trials. Hast Cent Rep.

[5] Saxman S (2014). Ethical considerations for outcome-adaptive trial designs: a clinical researcher’s perspective. Bioethics..

[6] Arksey H, O'Malley L (2005). Scoping studies: towards a methodological framework. Int J Soc Res Methodol.

[7] Benda N, Brannath W, Bretz F, Burger H, Friede T, Maurer W (2010). Perspectives on the use of adaptive designs in clinical trials. Part II. Panel discussion. J Biopharm Stat.

[8] Chappell R, Durkalski V, Joffe S (2017). University of Pennsylvania Ninth Annual Conference on Statistical Issues in Clinical Trials: where are we with adaptive clinical trial designs? (morning panel discussion). Clin Trials.

[9] Coffey C, Levin B, Clark C, Timmerman C, Wittes J, Gilbert P (2012). Overview, hurdles, and future work in adaptive designs: perspectives from a National Institutes of Health-funded workshop. Clin Trials.

[10] DellaCioppa G (2013). Executive roundtable on adaptive design trials identifies key factors that dramatically increase development efficiency and productivity.

[11] Dibao-Dina C, Caille A, Giraudeau B (2018). Heterogeneous perception of the ethical legitimacy of unbalanced randomization by institutional review board members: a clinical vignette-based survey. Trials..

[12] Dimairo M, Boote J, Julious S, Nicholl J, Todd S (2015). Missing steps in a staircase: a qualitative study of the perspectives of key stakeholders on the use of adaptive designs in confirmatory trials. Trials..

[13] Dimairo M, Julious S, Todd S, Nicholl J, Boote J (2015). Cross-sector surveys assessing perceptions of key stakeholders towards barriers, concerns and facilitators to the appropriate use of adaptive designs in confirmatory trials. Trials..

[14] Elsäßer A, Regnstrom J, Vetter T, Koenig F, Hemmings R, Greco M (2014). Adaptive clinical trial designs for European marketing authorization: a survey of scientific advice letters from the European Medicines Agency. Trials..

[15] Food & Drug Administration. Adaptive design clinical trials for drugs and biologics. Silver Spring: Center for Drug Evaluation and Research Center for Biologics Evaluation and Research; 2018.

[16] Guetterman T, Fetters M, Legocki L, Mawocha S, Barsan W, Lewis R (2015). Reflections on the adaptive designs accelerating promising trials into treatments (ADAPT-IT) process—findings from a qualitative study. Clin Res Regul Aff.

[17] Hartford A, Thomann M, Chen X, Miller E, Bedding A, Jorgens S, et al. Adaptive designs: results of 2016 survey on perception and use. Ther Innov Reg Sci. 2018:54(1):42–54. 10.1007/s43441-019-00028-y 32008237

[18] Jaki T (2013). Uptake of novel statistical methods for early-phase clinical studies in the UK public sector. Clin Trials..

[19] Legocki L, Meurer W, Frederiksen S, Lewis R, Durkalski V, Berry D (2015). Clinical trialist perspectives on the ethics of adaptive clinical trials: a mixed-methods analysis. BMC Med Ethics..

[20] Lin M, Lee S, Zhen B, Scott J, Horne A, Solomon G (2016). CBER’s experience with adaptive design clinical trials. Ther Innov Reg Sci.

[21] Meurer W, Legocki L, Mawocha S, Frederiksen S, Guetterman T, Barsan W (2016). Attitudes and opinions regarding confirmatory adaptive clinical trials: a mixed methods analysis from the Adaptive Designs Accelerating Promising Trials into Treatments (ADAPT-IT) project. Trials..

[22] Morgan C, Huyck S, Jenkins M, Chen L, Bedding A, Coffey C (2014). Adaptive design: results of 2012 survey on perception and use. Ther Innov Reg Sci.

[23] Quinlan J, Gaydos B, Maca J, Krams M (2010). Barriers and opportunities for implementation of adaptive designs in pharmaceutical product development. Clin Trials.

[24] Wang S (2010). Perspectives on the use of adaptive designs in clinical trials. Part I. Statistical considerations and issues. J Biopharm Stat.

