# Supplementary material for: Stakeholder perspectives on adaptive clinical trials: a scoping review
Source: Trials. 2020 Jun 17;21:539. doi: 10.1186/s13063-020-04466-0 (PMC7301522; doi:10.1186/s13063-020-04466-0)
Supplement: Supplementary file 1 — Additional file 1:Supplementary Table 1. Search terms. Supplementary Table 2. List of records screened and excluded due to not meeting inclusion criteria. Supplementary Table 3. List of full-text articles assessed but excluded due to not meeting inclusion criteria. [file 13063_2020_4466_MOESM1_ESM.docx]

**Supplementary Table 1: Search terms**

| (adaptive clinical trial) AND (perspectives) | (adaptive design) AND (perspectives) |
| --- | --- |
| (adaptive clinical trial) AND (attitudes) | (adaptive design) AND (barriers) |
| (adaptive clinical trial) AND (perception) | (adaptive design) AND (survey) |
| (adaptive clinical trial) AND (ethics) | (adaptive design) AND (ethics) |
| (adaptive clinical trial) AND (review board) | (adaptive design) AND (perception) |
| (adaptive clinical trial) AND (survey) | (adaptive design) AND (attitudes) |

**Supplementary Table 2: List of Records Screened and Excluded Due to Not Meeting Inclusion Criteria**

| Ethics and Practice: Alternative Designs for Phase III Randomized Clinical Trials | 1999 |
| --- | --- |
| A Conceptual Framework for Adaptive Preventive Interventions | 2004 |
| Methodological Developments vs. Regulatory Requirements | 2006 |
| The reassessment of trial perspectives from interim data—a critical view | 2006 |
| Clinical trials in children with Down syndrome: Issues from a cognitive research perspective | 2006 |
| Adaptive Allocation and Failure Saving in Randomised Clinical Trials | 2006 |
| Response-Adaptive Randomization for Clinical Trials with Continuous Outcomes | 2006 |
| FDA perspective on trials with interim efficacy evaluations | 2006 |
| FDA's Critical Path Initiative: A Perspective on Contributions of Biostatistics | 2006 |
| Adaptive two‐stage designs in phase II clinical trials | 2006 |
| Opening the Adaptive Toolbox | 2006 |
| Adaptive Dunnett tests for treatment selection | 2008 |
| Bayesian adaptive design for targeted therapy development in lung cancer-a step toward personalized medicine | 2008 |
| Adaptive designs for confirmatory clinical trials | 2009 |
| Adaptive Randomization for Multiarm Comparative Clinical Trials Based on Joint Efficacy / Toxicity Outcomes | 2009 |
| Good Practices for Adaptive Clinical Trials in Pharmaceutical Product Development | 2009 |
| FDA Draft Guidance on Adaptive Design Clinical Trials: Pfizer's Perspective | 2010 |
| Adaptive clinical trials for new drug applications in Japan | 2010 |
| Viewpoints on the FDA Draft Adaptive Designs Guidance from the PhRMA Working Group | 2010 |
| Clinical trials in ventilator treatment: current perspectives and future challenges | 2010 |
| Handbook of adaptive designs in pharmaceutical and clinical development | 2011 |
| Adaptive Trial Design: Could We Use This Approach to Improve Clinical Trials in the Field of Global Health? | 2011 |
| Dynamic treatment regimes for managing chronic health conditions: a statistical perspective | 2011 |
| Hierarchical Commensurate and Power Prior Models for Adaptive Incorporation of Historical Information in Clinical Trials | 2011 |
| How to improve the clinical development paradigm and its division into phases I, II and III | 2011 |
| Clinical trial design: Bayesian and frequentist adaptive methods | 2012 |
| Clinical Trial Design | 2012 |
| Adaptive Randomization for Clinical Trials | 2012 |
| Designing a pilot sequential multiple assignment randomized trial for developing an adaptive treatment strategy | 2012 |
| Adaptive and flexible clinical trials | 2012 |
| Adaptive Design Methods in Clinical Trials | 2012 |
| Paradigms for adaptive statistical information designs: practical experiences and strategies | 2012 |
| Adaptive clinical trials in oncology | 2012 |
| Phase II trial design with Bayesian adaptive randomization and predictive probability | 2012 |
| Re‐randomization tests in clinical trials | 2012 |
| Pathological complete response rates following different neoadjuvant chemotherapy regimens for operable breast cancer according to ER status, in two parallel, randomized phase II trials with an adaptive study design (ECTO II) | 2012 |
| Bayesian adaptive clinical trials: a dream for statisticians only? | 2012 |
| Adaptive clinical trial designs to detect interaction between treatment and a dichotomous biomarker | 2013 |
| Adaptive clinical trial designs with pre‐specified rules for modifying the sample size: a different perspective | 2013 |
| Targeting the SYK-BTK axis for the treatment of immunological and hematological disorders: recent progress and therapeutic perspectives | 2013 |
| Determining optimal sample sizes for multistage adaptive randomized clinical trials from an industry perspective using value of information methods | 2013 |
| Authors' response to ‘Adaptive clinical trial designs with pre‐specified rules for modifying the sample size: a different perspective | 2013 |
| Do Bayesian adaptive trials offer advantages for comparative effectiveness research? Protocol for the RE-ADAPT study | 2013 |
| New evidence-based adaptive clinical trial methods for optimally integrating predictive biomarkers into oncology clinical | 2013 |
| Adaptive Multivariate Global Testing | 2014 |
| Antibody Fc: linking adaptive and innate immunity | 2014 |
| Health related quality of life and patient reported symptoms before and during definitive radio(chemo)therapy using image-guided adaptive brachytherapy for locally advanced cervical cancer and early recovery — A mono-institutional prospective study | 2014 |
| Efficient and Ethical Response-Adaptive Randomization Designs for Multi-Arm Clinical Trials With Weibull Time-to-Event Outcome | 2014 |
| Patient-Centeredness in the Design of Clinical Trials | 2014 |
| The outcome of a multi-centre feasibility study of online adaptive radiotherapy for muscle-invasive bladder cancer TROG 10.01 BOLART | 2014 |
| Adaptive Designs for Confirmatory Clinical Trials with Subgroup Selection | 2014 |
| Response‐adaptive decision‐theoretic trial design: operating characteristics and ethics | 2014 |
| A comparison of Bayesian adaptive randomization and multi-stage designs for multi-arm clinical trials | 2014 |
| Spinal Cord Injury Neuroprotection and the Promise of Flexible Adaptive Clinical Trials | 2014 |
| The Brave New World of clinical cancer research: Adaptive biomarker-driven trials integrating clinical... | 2015 |
| Adding a treatment arm to an ongoing clinical trial: a review of methodology and practice | 2015 |
| Immunologic approaches to cancer prevention—current status, challenges, and future perspectives | 2015 |
| What have we learned from exceptional tumour responses?: review and perspectives | 2015 |
| An objective re-evaluation of adaptive sample size re-estimation: commentary on 'Twenty-five years of confirmatory adaptive designs' | 2015 |
| Interleukin-1 as a Common Denominator from Autoinflammatory to Autoimmune Disorders: | 2015 |
| Adaptive Crossover Design for Normal Responses | 2015 |
| Optimizing Trial Designs for Targeted Therapies | 2016 |
| Adaptive designs undertaken in clinical research: a review of registered clinical trials | 2016 |
| Twenty‐five years of confirmatory adaptive designs: opportunities and pitfalls | 2016 |
| Biomarker-Guided Adaptive Trial Designs in Phase II and Phase III: A Methodological Review | 2016 |
| Minimax and admissible adaptive two-stage designs in phase II clinical trials | 2016 |
| Sample Size Reassessment and Hypothesis Testing in Adaptive Survival Trials | 2016 |
| Antitumor dendritic cell–based vaccines: lessons from 20 years of clinical trials and future perspectives | 2016 |
| Optimal adaptive two‐stage designs for early phase II clinical trials | 2016 |
| Adaptive Design for a Confirmatory Basket Trial in Multiple Tumor Types Based on a Putative Predictive Biomarker | 2016 |
| Adapting drug approval pathways for bacteriophage-based therapeutics | 2016 |
| Participant preferences for pharmacologic chronic pain treatment trial characteristics: an ACTTION adaptive choice-based conjoint study | 2016 |
| PIPELINEs: Creating Comparable Clinical Knowledge Efficiently by Linking Trial Platform | 2016 |
| Opportunities and challenges for drug development: public-private partnerships, adaptive designs and big data | 2016 |
| Opportunities and challenges for drug development: public-private partnerships, adaptive designs and big data | 2016 |
| Adaptive Randomization of Veliparib–Carboplatin Treatment in Breast Cancer | 2016 |
| Clinical Perspectives on Targeting Therapies for Personalized Medicine | 2016 |
| A Bayesian adaptive dose-finding algorithm for balancing individual- and population-level ethics in Phase I clinical trials | 2016 |
| Methods for identification and confirmation of targeted subgroups in clinical trials: A systematic review | 2016 |
| A conceptual model for the development process of confirmatory adaptive clinical trials within an emergency research network | 2017 |
| Adaptive designs from a Data Safety Monitoring Board perspective: Some controversies and some case studies | 2017 |
| Adaptive prior weighting in generalized regression | 2017 |
| Sleep Apnea and Cardiovascular Disease: Lessons from Recent Trials and Need for Team Science | 2017 |
| Adaptive goal setting and financial incentives: a 2 × 2 factorial randomized controlled trial to increase adults’ physical activity | 2017 |
| Hypertrophic Burn Scar Research: From Quantitative Assessment to Designing Clinical Sequential Multiple Assignment Randomized Trials | 2017 |
| Early phase clinical trials of anticancer agents in children and adolescents -- an ITCC perspective | 2017 |
| Improving patient reported outcomes using item response theory and computerized adaptive testing | 2017 |
| Administration of Rhodiola kirilowii Extracts during Mouse Pregnancy and Lactation Stimulates Innate but Not Adaptive Immunity of the Offspring | 2017 |
| Can Lokomat therapy with children and adolescents be improved? An adaptive clinical pilot trial | 2017 |
| Strength of baseline inter-trial correlations forecasts adaptive capacity in the vestibulo-ocular reflex | 2017 |
| Bayesian Randomized Clinical Trials: From Fixed to Adaptive Design | 2017 |
| Development of an adaptive, personalized, and scalable dementia care program: Early findings from the Care Ecosystem | 2017 |
| Bayesian doubly adaptive randomization in clinical trials | 2017 |
| Efficient confidence limits for adaptive one-arm two-stage clinical trials with binary endpoints | 2017 |
| Subgroup selection in adaptive signature designs of confirmatory clinical trials | 2017 |
| The changing landscape of data monitoring committees—Perspectives from regulators, members, and sponsors | 2018 |
| Biopharmaceutical Applied Statistics Symposium Volume 2 Biostatistical Analysis of Clinical Trials | 2018 |
| Rationale and Design of an Adaptive Phase 2b/3 Clinical Trial of Selepressin for Adults in Septic Shock Selepressin Evaluation Programme for Sepsis-induced Shock-Adaptive Clinical Trial | 2018 |
| Critical concepts in adaptive clinical trials | 2018 |
| Interpreting the Regulatory Perspective on Adaptive Designs | 2018 |
| Adaptive crossover designs for assessment of symptomatic treatments targeting behaviour in neurodegenerative disease: a phase 2 clinical trial of intranasal oxytocin for frontotemporal dementia (FOXY) | 2018 |
| Key design considerations for adaptive clinical trials: a primer for clinicians | 2018 |
| Biopharmaceutical Applied Statistics Symposium | 2018 |
| Adaptive designs in clinical trials: why use them, and how to run and report them | 2018 |
| Treatment of bipolar depression with minocycline and/or aspirin: an adaptive, 2×2 double-blind, randomized, placebo-controlled, phase IIA clinical trial | 2018 |
| A new design strategy for hypothesis testing under response adaptive randomization | 2018 |
| Adaptive design in surveys and clinical trials: similarities, differences and opportunities for cross‐fertilization | 2018 |
| Lessons learned from IDeAl - 33 recommendations from the IDeAl-net about design and analysis of small population clinical trials | 2018 |
| Treatment evaluation for a data‐driven subgroup in adaptive enrichment designs of clinical trials | 2018 |
| Learning health systems, clinical equipoise and the ethics of response adaptive randomisation | 2018 |
| Adaptive design clinical trials: a review of the literature and ClinicalTrials.gov | 2018 |
| Practical characteristics of adaptive design in phase 2 and 3 clinical trials | 2018 |
| Addressing heterogeneity in the design of phase II clinical trials in geriatric oncology | 2018 |
| Optimized adaptive enrichment designs | 2019 |
| Clinical Equipoise and Adaptive Clinical Trials | 2019 |
| Adaptive immune response to Clostridium difficile infection: A perspective for prevention and therapy | 2019 |
| The Case for Adaptive Neuromodulation to Treat Severe Intractable Mental Disorders | 2019 |
| Outcome-adaptive randomization in clinical trials: issues of participant welfare and autonomy | 2019 |
| When to keep it simple - adaptive designs are not always useful | 2019 |

**Supplementary Table 3: List of Full-Text Articles Assessed but Excluded Due to Not Meeting Inclusion Criteria**

| Adaptive Designs from the Viewpoint of an Academic Biostatistician | 2006 |
| --- | --- |
| Challenges in Implementing Adaptive Designs: Comments on the Viewpoints Expressed by Regulatory Statisticians | 2006 |
| A Consultant's Perspective on the Regulatory Hurdles to Adaptive Trials | 2006 |
| Understanding the FDA Guidance on Adaptive Designs: Historical, Legal, and Statistical Perspectives | 2010 |
| Efficiency perspectives on adaptive designs in stroke clinical trials | 2011 |
| Regulatory Perspectives on Multiplicity in Adaptive Design Clinical Trials throughout a Drug Development Program | 2011 |
| Benefits, challenges and obstacles of adaptive clinical trial designs | 2011 |
| Adaptive trial designs: a review of barriers and opportunities | 2012 |
| Adaptive Trials in Clinical Research: Scientific and Ethical Issues to Consider | 2012 |
| Adaptive designs: The Swiss Army knife among clinical trial designs? | 2017 |
| Can emergency medicine research benefit from adaptive design clinical trials? | 2017 |
| Response‐adaptive clinical trials: case studies in the medical literature | 2017 |
| The Real‐World Ethics of Adaptive‐Design Clinical Trials | 2017 |
| Adaptive designs in clinical trials: from scientific advice to marketing authorisation to the European Medicine Agency | 2018 |
| Data monitoring in clinical trials: a practical perspective | 2019 |
